# Multiperspectivity as a Process of Understanding and Reflection: Introduction to a Model for Perspective-Taking in Geography Education

**DOI:** 10.3390/ejihpe11020038

**Published:** 2021-06-10

**Authors:** Dina Vasiljuk, Alexandra Budke

**Affiliations:** Institute for Geography Education, University of Cologne, Gronewaldstraße 2, 50931 Cologne, Germany; alexandra.budke@uni-koeln.de

**Keywords:** perspective-taking, multiperspectivity, using different perspectives, competency models, decision making, competency assessment

## Abstract

Perspective-taking is an essential competency because it enables a better understanding of complex issues or conflicts with various actors and different points of view involved. However, no competency model for perspective-taking has been provided in geography education to date, which is why a respective model was developed in this study. The model was then applied by analysing 28 articles from four practice journals of geography education from German-speaking countries. This analysis focused on the dimensions of the perspective-taking competencies that were required by respective tasks within the lesson concepts. The results show that the tasks hardly promoted competence-oriented geography teaching in terms of perspective-taking. Therefore, the competency model could be a suitable tool for analysing and developing teaching materials that implement perspective-taking.

## 1. Introduction

Changing climate, water crises, ageing population or income inequality are examples of the contemporary, highly complex challenges facing today’s society [1]. The complexity of these issues or problems shows that there is no incontrovertible right or wrong opinion and solution, but rather different points of view which lead to different truths [2,3] (p. 14). Therefore, an aim in geography education in Germany is the encouragement of multi-perspective thinking [4] (p. 2), so that the students are able to deal with these complex developments by “identify[ing] the interests of different actors, look[ing] at a situation from the point of view of different people affected and thereby practic[ing] the skill of changing perspectives” [4] (p. 22). Perspective-taking is a fundamental ability in today’s society [5] (p. 2), and it is important for children’s learning processes to reduce egocentrism [6]. According to Duncker [3] (p. 18), perspective-taking is relevant in various situations, such as when positions do not match in discussions, understanding is demanded, empathy is needed, or when differences of opinions must be accepted or at least tolerated. Being able to “identify and evaluate such points of view greatly enhances our own decision-making processes” [7] (p. 1). Different points of view lead to different truths; therefore, the deconstruction of perspectives in the context of perspective-taking is also a necessary skill and, moreover, an educational standard in geography that is important for democratic participation in society [8] (p. 273). Therefore, implementing perspective-taking as a competency in geography lessons can contribute not only to the tolerance of ambiguity, but also to the enhancement of our own decision-making processes and democratic participation. Thus, it should not only be considered in lessons, but be guided and encouraged by tasks. Perspective-taking is a “complex and multifaceted sociocognitive process” [5] (p. 2); therefore, it is important to subdivide the process into dimensions so that students can apply it in a meaningful way. Although perspectivism and thus also perspective-taking are already attributed to Nietzsche, there are very few theoretical approaches in the field of geography education that discuss the dimensions of perspective-taking.

Therefore, we propose a theoretical competency model for perspective-taking in geography education in this study. We used this model to analyse the tasks of 28 articles from four journals focused on geography education from German-speaking countries, in which the authors used perspective-taking in their lesson concepts. With the help of the competency model, we applied qualitative content analysis to differentiate the tasks in the articles and to evaluate them with regard to the competency of perspective-taking. Therefore, this article aims to answer the following research questions:

- How can we model perspective-taking competency?
- What competency dimensions of perspective-taking are enhanced by the tasks in lesson concepts?

First, we will present the theoretical background. In the second section, we will explain our model of perspective-taking, followed by a description of the methodological approach. Finally, we will present the empirical results and discuss the potential of our model to help design perspective-taking tasks for geography lessons.

## 2. Competency Model of Perspective-Taking in Geography Education

In the following sections, we will first present perspective-taking through various concepts, theories and models, and discuss in what ways perspective-taking is an important competency in geography education. Then, we will present our model for perspective-taking for geography education in more detail.

### 2.1. Theoretical Background: Perspective-Taking as a Competency

Perspective-taking is not a new topic. Perspectives and points of view were already discussed in perspectivism. Nietzsche described perspectives as standpoints, and therefore as perceptions of reality so that humans can schematise, regulate and simplify the chaos of the world, because this is what humans’ practical needs require. However, the human being cannot overcome perspectivity, because their existence causes it. At the same time, a human will always think of their simplification as the correct interpretation. Therefore, awareness of perspective-taking is necessary, so that, for example, a complex topic can be better understood. Based on the human way of being, which does not allow considering all perspectives at the same time, it is important that students understand and reflect that an absolute view on an issue is not possible [9,10] (p. 267). In geography education, *reflexion* is therefore a competency to gain a critical distance to, e.g., one’s own thinking, knowledge or skills through perspective-taking [11,12] (p. 25). According to Piaget [13,14], children gradually learn to distance themselves from their own point of view and take on other viewpoints instead. He describes this process as *decentring*. Edelstein and Keller [15,16] argued that the process should be divided into *perspective differentiation* and *perspective coordination*. Therefore, “perspective-taking is taken to represent the formal structure of coordination of the perspectives of self and other as they relate to the different categories of people’s naive theories of action […] The ability to differentiate and coordinate the perspectives of self and other thus is a necessary condition both in the development of making socio-moral meaning and in the actual process of solving situations of conflicting claims” [16] (p. 96). Therefore, the deconstruction of perspectives is an important ability and a key qualification in geography for participation in a democratic society [8,17].

Depending on the type of perspective-taking, a distinction is made in psychology between the *visual*, *affective* and the *cognitive* perspective-taking [18] (p. 471). According to Gollin and Sharps [19] (p. 435), *visual perspective-taking* “involves the understanding that one’s view of a given scene may differ from the view of other observers”: so-called *spatial egocentrism* [20,21] (pp. 263–265). *Affective perspective-taking* is the ability to understand the feelings and emotions of another person [5,22]. *Cognitive perspective-taking* is the ability to understand the other person’s thoughts and being able to assess the person’s behaviour [5,23]. Therefore, according to Edelstein and Keller [15,16], perspective-taking is fundamental for human interaction and communication. With the help of perspective-taking, social actions can be coordinated, and a common understanding of a situation can be leveraged. The understanding of social interaction and social realities is also a fundamental part of today’s geography [24], which is why students should be able to apply this ability in geography lessons as well.

Referring to Ashby and Lee [25,26], Hartmann and Hasselhorn [27] (p. 264) define *historical perspective-taking* (HPT) as a competency which means “knowing that certain historical agents or groups had particular perspectives on their world, and being able to see how that perspective would actually have affected actions in different situations”. In this context, it is important to understand that actions of historical actors were significantly influenced by different values and beliefs. Students who accomplish historical perspective-taking will not only “benefit in terms of historical understanding but will also be better able to cope with the present world” [27] (p. 264). Understanding the actors in the context of existing values is also important in geography education. Therefore, an aim in the German educational standards in geography is that students can identify the interests of different actors, look at a situation from standpoints of different people, and can thereby practise the skill of perspective-taking [4] (p. 22). According to Reinhardt [28], perspective-taking is an essential competency for dealing with conflicts and complex issues. Furthermore, perspective-taking is essential for political judgement in democratic education because perspectives are results from a different social environment or social context. Therefore, to understand and to evaluate an actor’s perspective, the analysis of actors is important, too [29]. By analysing, for example, the *actor scale*, *category* and *type*, the students can better understand the actor’s ability to act as well as the actor’s assertiveness of their interests [29]. Besides analysing the actors chosen for perspective-taking, it is also relevant in problem-oriented geography lessons to compare different perspectives with defined criteria so that students can identify similarities and differences between the perspectives [30] (p. 4).

Being able to understand different points of view on an issue by perspective-taking is essential for a reflective statement. Additionally, students learn to abstract from one’s own position, and they can learn to understand the different value orientations and judgements of others [31,32]. Geographical education, in particular, contributes to an understanding of values, ways of life and other cultures [33]. Therefore, perspective-taking is an important skill for understanding the topic in its complexity on the one hand, and for promoting one’s own tolerance on the other [34]. In the field of geography education, Rhode-Jüchtern [35] (pp. 4–5) considers perspective-taking as a method to work out—depending on the topic and problem—differences regarding the points of view, which is why perspective-taking is a way of asking to foster a certain recognition, judgement or action in geography education. Furthermore, in educational standards in geography, perspective-taking is important for the evaluation of competence. Students should be able to evaluate facts and arguments on the basis of criteria [4] (pp. 22–24).

As already shown, perspective-taking is assigned to evaluation competence in the educational standards for geography; however, evaluation is not the only dimension of perspective-taking, as other disciplines have pointed out. Perspective-taking is about more than that. It can help students not only to understand other perspectives and to develop their own opinion, but also to question their own knowledge [36].

Perspective-taking is considered an important method in geography education [35]. Furthermore, geography textbooks have been analysed in terms of how multiperspectivity is implemented [37], or how multiperspectivity is used in planning tasks [38]. Additionally, role plays with multiperspectivity as a guiding concept were analysed in various exercises [39].

However, perspective-taking as a competency in geography education has not yet been discussed. Therefore, we propose a competency model for perspective-taking in geography education in the following section. The aim of this model is to show the dimensions of perspective-taking and thus to also show the complexity of the competency.

### 2.2. Competency Model for Perspective-Taking in Geography Education

This article aims to propose a competency model for perspective-taking, because different theoretical concepts have been discussed in previous research but no competency model for perspective-taking has been provided in geography education. To promote perspective-taking in geography lessons, a theoretical model has to be developed first, in which central dimensions describe the competence of perspective-taking [40] (p. 183). Therefore, we explain our competency structure model for geography education in the following sections.

Existing models for perspective-taking developed for other disciplines cannot be fully adapted for geography education. For example, Selman [41], following Kohlberg’s step model for moral awareness [42,43], developed a model for *social perspective-taking*. However, this is a social–cognitive development model and does not deal with the ability to evaluate issues or conflicts in different disciplines, nor with the reflection of the perspectives that have been taken.

In the field of biology education, perspective-taking is implemented into a theoretical model of *moral judgement* [34]. However, perspective-taking is only a small part of the judgement of bioethical problems. Perspective-taking is not considered as a single competence. Instead, it is directly linked to the areas of assessment and reflection. The competency of perspective-taking is not differentiated, and the complexity is not further explored. Therefore, this assignment does not meet the requirements of perspective-taking.

Intercultural competence perspective-taking is used to take over another person’s perspective so that distancing from one’s own views is possible [44] (p. XII). Bredella et al. [44] proposed the approach of differentiation between an *inside* and an *outside perspective*. Inside perspective means “to see the other’s culture through the eyes of the members of that same culture […] outside perspective means that the other’s culture is always seen through one’s own eyes and understood based on one’s unavoidable preconceptions” [45] (p. 2778). In this approach, Bredella et al. [44] (2000) identified different forms of relationality between inside and outside perspectives. It shows, for example, that students can accept arguments from other perspectives and that students can become aware of the similarities and differences of cultures by using perspective-taking. The approach already shows how complex perspective-taking is, and that a differentiation of the competency is necessary. However, the approach to intercultural understanding does not consider other sub-competencies that are necessary for perspective-taking. The approach does not show how students should implement perspective-taking. Furthermore, it is not explained what other competencies the students need for perspective-taking.

In the field of history education, Conrad [46], following the model for *historical empathy* by Asby and Lee [25], developed a model that contains the three competencies of perspective-taking, explanation and evaluation, with the aim of promoting historical awareness. However, the perspectives of the actors were not analysed with different criteria, nor spatially defined or reflected, as would be relevant for the geographical context in order to understand the relevant issue or conflict. The primary aim was to reconstruct and understand the historical perspectives using the three competencies. Nevertheless, the model shows that perspective-taking can vary greatly in its complexity.

**Figure 1:**
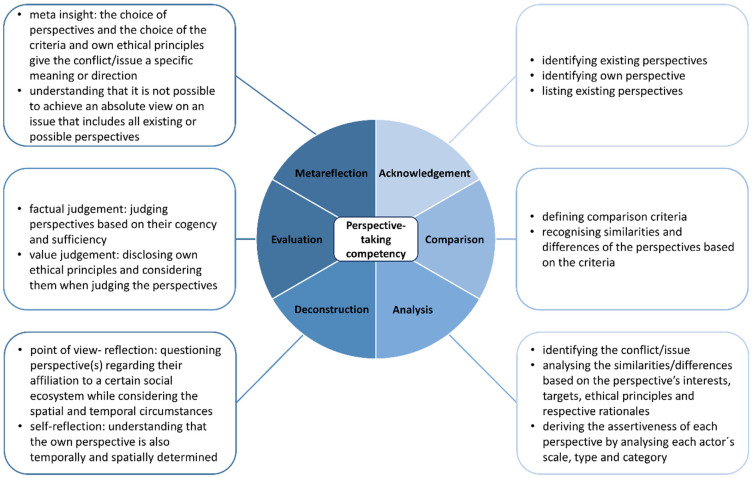
Competency structure model for perspective-taking (own design).

Based on the theories, concepts and models that have been introduced, we now propose a competency model for perspective-taking in geography education. The normative model is divided into six dimensions: *acknowledgement*, *comparison*, *analysis*, *deconstruction*, *evaluation* and *metareflection* (Figure 1). In order to illustrate the dimension, we will use the example of a rainforest with the overall question: *Should the rainforest be exclusively protected and not used for agriculture?*

We will introduce the dimensions using two typical perspectives on the issues that are often presented in geography journals, lesson proposals or textbooks [47,48,49]: the *small farmer* and the *environmentalists* (who is a representative of an European environmental institution).

**Dimension—acknowledgement:** In the acknowledgement dimension, students should understand that there are other perspectives besides their own [13,14,44].

Therefore, students should be able to identify that there are various perspectives for a geographical issue or conflict in relation to the question and list these perspectives.

In our example, students should be able to identify and to list the perspective of the *small farmer* and the *environmentalist* and also be able to identify their own perspective. Furthermore, they should be able to reproduce the overall question (*Should the rainforest be exclusively protected and not used for agriculture?).*

**Dimension—comparison:** In the comparison dimension, students should be able to compare the perspectives of the actors to understand an issue or conflict [30,44]. Therefore, students should be able to recognise similarities and differences in the respective views of the actors. They compare the views of the respective actors based on defined or self-chosen criteria that correspond to an overall question [30].

In our example, students should compare the perspectives (*small farmer* and *environmentalist*) to an overall question (*Should the rainforest be exclusively protected and not used for agricultural*?). For our example, we define two criteria: *environmental use* and *environmental protection*. Subsequently, we briefly summarise the perspectives per criterion as examples.

Criterion—environmental use: The small farmer uses the rainforest to feed their family and sometimes sells the surplus crops. The environmentalist supports the ban on commercial uses of the rainforest. The perspectives consider the uses of the rainforest to be partially different.

Criterion—environmental protection: The small farmer thinks that the environment is important but nonetheless prioritises their family’s basic needs. The environmentalist wants to protect the rainforest so that the habitat of various animals is not destroyed. Both perspectives want to protect the rainforest (similarity), but they have different direct dependencies on the rainforest.

Based on the very simple criteria, it can already be seen that there are differences between the perspectives (commercial use of the rainforest), but also some hints of similarities (both actors think that environmental protection is not unimportant).

**Dimension—analysis:** In the analysis dimension, it is important that students are able to identify the issues or conflicts that result from the different perspectives [28]. Thus, they should be able to understand the other person’s thoughts [5,23] and feelings [5,22]. In order to understand and structure the feelings and thoughts, criteria are important. Therefore, the students should be able to analyse the arguments of the different actors, e.g., regarding the perspective’s interests, targets, ethical principles and respective rationales [27,29] to be able to answer the overall question.

In our example, the aim of the environmentalist is to protect the animals in the rainforest and to inform others about the destruction of the rainforest and its consequences, such as climate change (interest and targets). The environmentalist also works with local farmers to develop collaborative solutions for rainforest use (ethical principles and rationales). The small farmer, on the other hand, wants to feed their family and earn some money from surplus crops (interest and targets). The rainforest is important, but global climate change, for example, is not as important as the daily food the family needs (interest and ethical principles). The small farmer joins concepts of environmental institutions (rationales).

In addition to the perspective’s interests, targets, ethical principles and respective rationales, the analysis must also consider the actors’ ability to act and their assertiveness abilities so that students can understand the developments of the issue or conflict [27,28]. Therefore, it is important for students to consider the *actor scale*, the *actor category* and the *actor type* when taking a perspective [29].

Each perspective of an actor can be assigned to an actor scale (local, regional, national or global). The actor scale thus describes, on the one hand, the scale on which actors view a conflict and, on the other hand, the scale on which actors can primarily act. In addition, actors can be divided into two types of actors, which describe the type of viewing. A *direct actor* corresponds to a particular person (e.g., politicians, residents, environmentalists), so that an issue or conflict is viewed from the perspective of a particularly acting person. In the case of an *indirect actor*, the consideration is subject-specific (e.g., economic, social, environmental). Furthermore, direct actors can be divided into two types of actors: the *individual actor* type (e.g., resident) and the *complex actor* type (e.g., representative of a company, political decision-maker). Individual actors pursue their own interests, whereas complex actors are bound to the interests of the respective institution (e.g., companies or state institutions). The action of actors and the assertiveness of their interests often depend on the actor scale and actor type.

In our example, the small farmer is a local actor, the environmentalist is a global actor (actor scale). The small farmer considers the issue locally and is only able to act locally. The environmentalist, on the other hand, considers the conflict globally and is able to act globally because of affiliation with the international institution. Both actors are direct actors (actor category). The small farmer is an individual actor (actor type), and the environmentalist is a complex actor (actor type), because they are a representative of an international institution.

In summary, with the analysis of the actors, it can be assumed that the environmentalist, in contrast to the small farmer, is more likely to assert the interests of the institution.

**Dimension—deconstruction:** Perspectives are constructs [9,10]; therefore, decentring [13,14] and deconstruction are necessary [8,17] to gain a critical distance from one’s own thinking [11,12].

Therefore, the students should be able to develop a deconstruction that includes a *point of view reflection* and a *self-reflection* to answer an overall question. The deconstruction can help to question the perspectives more precisely, considering spatial and temporal circumstances, and can also help to identify contradictions. Therefore, the students should be able to dissect the perspective without interpreting the perspectives.

The first deconstruction is the point of view reflection, which is a critical reflection of the actor’s point of view that was chosen for perspective-taking. This means that the perspective is questioned regarding their affiliation to a certain social ecosystem (for example, concerning its interests, targets, ethical principles, assertiveness and relationships to other actors). The second deconstruction is self-reflection: a critical reflection of one’s own perspective. This means that one’s own opinion, as well as thoughts and actions, are examined. Therefore, one’s own perspective is also questioned, regarding affiliations to a certain social ecosystem, which is then compared with other perspectives.

We will explain the first deconstruction in more detail. In our example, the environmentalist is trying to protect the rainforest from further exploitation (interest). They are part of a European environmental institution and can take a more long-term view of the issue because they are not directly dependent on the rainforest (spatial and temporal circumstances). They recognise a complex relationship between the rainforest and global climate change. Therefore, they try to achieve a change on the local level with global financial resources from the international institution (targets, assertiveness). This perception and their social actions correspond to their construction of the current issue. The environmentalist considers the rainforest as an important source of life, but ignores the livelihood of the small farmer (an example of a contradiction). Hence, the environmentalist mostly ignores the needs of the small farmer in favour of the environment because they have other priorities in the issue.

This does not mean that the perspective of the environmentalist is worse than that from of small farmer, but it is a (Eurocentric) perspective on the issue and thus one perspective among many possible perspectives.

**Dimension—evaluation:** As already shown, perspective-taking is assigned to evaluation competence in geographical educational standards in Germany [4] and for moral judgement [31,34]. To develop a valuable evaluation, judging and assessing must be distinguished. When judging, students need to check statements, proposals or actions regarding their validity based on a set of criteria without presenting their own opinion. When assessing, the students must also judge statements, proposals or actions based on ethical principles. This way, their judgement is developed ethically through presenting their own opinion [4]. It is important to distinguish both from each other because they refer to different aspects of normative justifications [50] (p. 6). This is why it is also important to make a difference between *factual judgements* (*Sachurteil*) and *value judgements* (*Werturteil*) [51] (p. 28).

In this way, students will be able to answer an overall question based on two judgements (factual judgement and value judgement). Through the factual judgement, the students have to decide which arguments and criteria they want to use when forming their judgement. Afterwards, they judge the perspectives based on their cogency and sufficiency. Through the value judgement, the students have to decide which arguments of the actors they want to use. Finally, they disclose their own ethical principles and consider them when judging the perspectives. 

For example, with the factual judgement, students could judge that it is not possible to completely forbid the use of the rainforest (due to cogency and sufficiency of the argument from the environmentalist). However, regulations and laws can be adopted to protect the rainforest, so that externalities, for example, are punished more strictly. If students have a very strong environmental protection attitude, they may arrive at a value judgement to completely prohibit the use of the rainforest.

The explanations show that despite the factual judgement, the value judgement can be quite different. Therefore, it is important to distinguish the judgements so that they are not mixed in the explanation.

**Dimension—metareflection:** The metareflection leads to the understanding that it is not possible to achieve an absolute view on an issue that includes all existing or possible perspectives [9,10].

Students should understand that each perspective is just a single view from a fixed point, which can change depending on various factors. The students are able to consider this conclusion when answering an overall question.

In our examples, students should understand that both perspectives are only excerpts of reality that are temporally and spatially determined. The environmentalist considers the problem very ecologically, while the small farmer thinks of their basic needs, which must be covered so that they and their family can survive. Therefore, the students should understand that an issue could appear differently if other perspectives are chosen.

**Figure 2:**
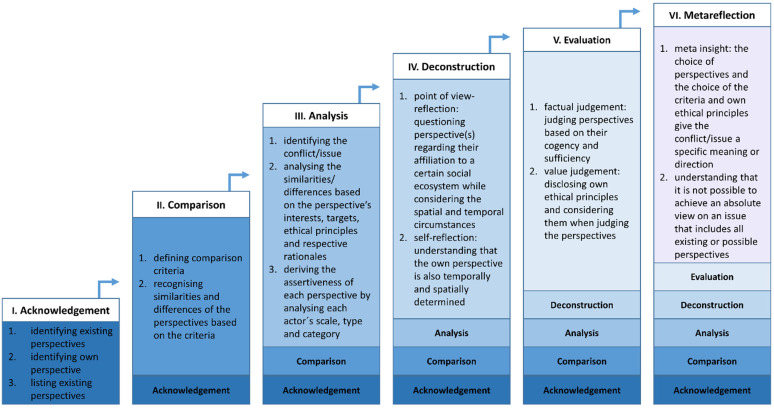
Competency model for perspective-taking with increasing competency (own design).

In addition, the assumption can be made that the dimensions could be interconnected and built on each other (Figure 2).

A metareflection may not be successfully developed without a factual and a value judgement, because both could be necessary to lead students to the finding that several perspectives can be valid and contrasting at the same time, and that these given perspectives are just a selection of theoretically endless possibilities.

In turn, the evaluation of the perspectives in terms of cogency and sufficiency via a factual and a value judgement should consider the perspectives’ spatial and temporal circumstances as well as their affiliation to a certain social ecosystem. Therefore, thorough evaluation may require a preceding deconstruction.

The deconstruction, on the other hand, benefits from a detailed analysis implemented at the forefront. This way, the perspectives’ interests, targets, ethical principles and rationales are present, and the assertiveness of each perspective regarding scale, type and category of the respective actor is already derived and analysed, which could help students considerably with the point of view reflection as well as with the self-reflection.

To analyse the different perspectives and to identify the conflict in more detail, a comparison which has already been implemented might be helpful for students as well, so that analysis of the similarities and differences of the perspectives is based on a defined selection of comparison criteria.

In conclusion, for a subsequent comparison of different perspectives, the identification of more than one perspective is certainly an essential acknowledgement.

In this case, further levels per dimension would have to be distinguished in order to be able to prove an increasing competency. However, this is not the aim in the context of this article, and should therefore only be considered as a footnote.

In summary, using perspective-taking in geography education is a complex challenge, because the competency of perspective-taking must be practised with students [52,53]. Therefore, tasks that instruct perspective-taking are a basic requirement.

Our competency model shows the different dimensions of perspective-taking that are used as a basis for the analysis of the articles and the tasks from the practice journals. The aim is to analyse which sub-competencies are enhanced by the students in perspective-taking, which is described in more detail in the following sections.

## 3. Methodology

In this article, we present an analysis of 28 articles from four practice journals focused on geography education from German-speaking countries to study which competencies are promoted through perspective-taking. The publications had to fulfil three criteria, which we will point out and explain.

First criteria (title): At least one of the following terms had to be used in the title or subtitle of the article: *perspective*, *perspectives*, *perspective*-*taking*, *multiperspectivity*, *multi-perspectivity*, *perspectivism*, *taking on perspective*, *understanding of others*, *intercultural understanding*, *interculturality*, *role taking*, *role play*, *role-playing game* and *taking the role of the other*. The choice of the title shows that multiperspectivity is the focus for the author in the lesson concept. We added role play and role-playing game to the search because, according to Silbereisen [54] (p. 64), this is a method that can help develop the understanding of social perspectives. Articles by authors who used the term “perspective” as a future prospect and did not deal with different points of view were not included in the analysis.

Second criteria (explanation): The authors’ didactic explanations of the topic showed which targets they pursued with multiperspectivity and how they defined the term.

Third criteria (task): The lesson concepts had to include tasks because they gave detailed instructions for perspective-taking and could also provide information for the analysis of the didactic targets.

There was no limitation in terms of publishing dates. Authors who had published the same article in two journals were only considered once for the analysis. In such cases, the article published first was selected for the analysis. A total of 28 articles matched these criteria. They were published between 1996 and 2017 in the practice journals of geography education *Geographie aktuell & Schule*, *GW-Unterricht*, *geographie heute* and *Praxis Geographie*.

We analysed the articles based on qualitative content analysis according to Mayring [55] and Kuckartz [56]. The classification of the analysis categories was inductive–deductive and, using the method outlined by Kuckartz [56] (pp. 100–115), the analysis process of the articles comprised seven phases. The first phase covered the initial work with the text including memos and case summaries and enabled the development of the main thematic categories (Phase 2) so that the data could be coded (Phase 3). In the fourth phase, the text passages assigned to the main category were summarised. This enabled us to differentiate and systematically approach the analysis as well as to create subcategories (Phase 5). In conclusion, a further coding process took place in which all selected articles were coded using the main and sub-categories. The seventh phase followed the last coding process in which both qualitative and quantitative analyses were implemented.

In the following section, the developed categories of the analysis are briefly introduced.

*Perspective-taking as a competency:* In this main category, we analysed which competencies were promoted according to the competency model for perspective-taking (Figure 1). We distinguished whether the perspective-taking competency was promoted *implicitly* or *explicitly* by the authors. In this context, *implicit perspective-taking* means that the single dimensions of perspective-taking (*acknowledgement*, *comparison*, *analysis*, *deconstruction*, *evaluation*, *metareflection*) were not explicitly demanded in the tasks. Instead, perspective-taking could merely be interpreted or was only explained in the didactic comments. Mentz [57] (pp. 22–25), for example, demanded a comparison of the arguments of the different actors in the didactic comments from the students but not explicitly in the tasks. It is therefore an implicit comparison and thus implicit perspective-taking (dimension comparison in Figure 1). 

In contrast, *explicit perspective-taking* means that the single dimensions of perspective-taking (*acknowledgement*, *comparison*, *analysis*, *deconstruction*, *evaluation*, *metareflection*) were explicitly demanded of the students in the tasks, as in the following example: “Compare the assessment regarding housing satisfaction and life prospects from the interviews” [58] (p. 57, own translation). The action verb “compare” can be assigned to the dimension comparison of the competency model (see Figure 1). This action verb explicitly requires the comparison of similarities and differences of the perspectives [4]. The task does not have to be interpreted further by the students, because the action verb gives them a clear instruction.

By analysing the tasks, we were able to determine and verify whether the authors required explicit perspective-taking or not. If a criterion of a dimension of the competence model was already achieved, it was evaluated as promoting the sub-competence of perspective-taking. Additionally, we considered the dimension deconstruction separately in the analysis (*point of view reflection* and *self-reflection*).

It should be noted that perspective-taking functioning as the material’s didactic focus did not occur in all analysed articles, and was only integrated as a smaller part in others. In some articles, perspective-taking was only used for the respective practice phase, as seen in this comment: “The final role play (M9) is a repetition of important contents of the lesson sequence and should lead the students to a multi-perspective approach and to an own well-founded evaluation of the problem” [59] (p. 31, own translation). Therefore, when identifying the dimensions of perspective-taking (see Figure 1), we only considered tasks that were directly related to perspective-taking.

## 4. Results and Discussion

In the following section, we present and discuss the results of applying the model to the 28 selected articles. The structure of this section is aligned with the competency model (see Figure 1).

**Figure 3:**
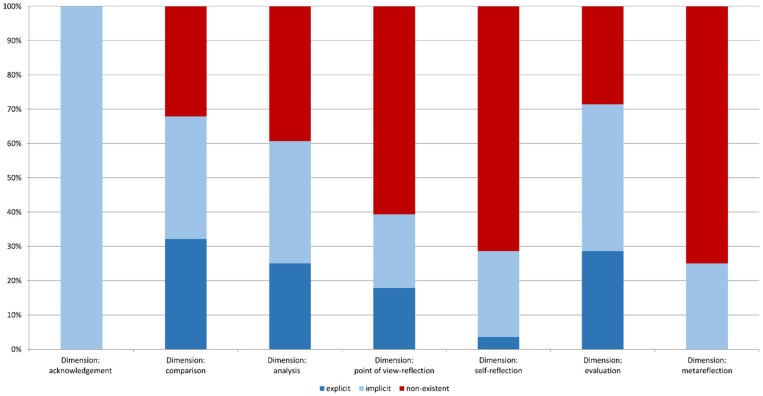
Implementation of the perspective-taking competency (explicit and implicit) in 28 analysed articles. Each article was assigned to every dimension (own elaboration).

The results show which dimension of the competency model is promoted by the authors in their lesson concepts (see Figure 3). We determined whether the respective competencies were promoted implicitly or explicitly (see Section 3).

An important finding for all dimensions is that only 15 of the 28 articles analysed contained an overall question regarding the conflict or the issue. Furthermore, Figure 3 shows that the explicit promotion of competencies for perspective-taking was not always included in the analysed articles. Nine of the 28 articles did not fulfil a single criterion of the dimensions (Figure 1) of explicit competency promotion. Thus, in nine lesson concepts (32%), no task could be identified that explicitly instructed how the students should accomplish perspective-taking. Therefore, it can be discussed whether the competency for perspective-taking can be promoted sufficiently because there are no tasks in the authors’ lesson concepts that explicitly describe which dimensions the students should adopt to achieve this. It cannot therefore be assumed that the students will be able to complete the perspective-taking holistically.

We now present the results for each dimension of perspective-taking competency and discuss them in more detail.

**Dimension: Acknowledgement**

When analysing the dimension *acknowledgement*, all selected articles implicitly corresponded to this dimension (see Figure 3), meaning that students had to choose a perspective and then consider other perspectives or roles in a discussion, such as in the following example: “Develop one role card each based on the former lesson contents. Make a list of bullet points to prepare a short presentation for a discussion and select “your” discussion participant” [59] (p. 34, own translation). In other cases, such as this example, the students had to consider a conflict from different perspectives: “Develop an argumentation (thesis, argument, example, conclusion) from both the refugee’s perspective and the host country (e.g., Turkey, Jordan, Germany)” [60] (p. 25, own translation). In all cases, students should identify that there are several perspectives on a conflict or on an issue. The authors do not expect students to list the individual perspectives explicitly in these cases.

Therefore, the assumption can be made that the perspectives are already apparent from the tasks or the materials and thus do not need to be explicitly listed. However, to be able to identify the actors in the issue or conflict, an explicit listing of the perspectives may be helpful. Using such an approach enables students to become aware of the existing actors in the space. For example, based on the explicit acknowledgement, the number of individual and complex actors could then be determined more easily in the third dimension of perspective-taking, and their interests could be analysed regarding their similarities and differences.

**Dimension: Comparison**

In the lesson concepts analysed, *comparison* was implemented (implicitly or explicitly) in 68% of the articles (see Figure 3). However, contrary to what was assumed, the required comparison was not always directly recognisable in the tasks, as shown in the following example: “In Task 3 you have observed the economical, ecological and social fields of the triple bottom line. To understand Benidorm entirely, you must relate these fields to each other” [61] (p. 37, own translation). Thus, the relationship between these fields can only be deduced if both similarities and differences are found, which is a comparison. Overall, in only 32% of the analysed articles, the comparison of perspectives was explicitly required through the tasks (see Figure 3). In only 18% of the analysed articles, comparison had to be performed based on defined criteria. The students were not asked in any task to define their own criteria to undertake the comparison.

Overall, it can be assumed that because of the didactic explanations or the implicit tasks, the comparison of perspectives is considered to be self-evident for the authors and is therefore not always explicitly asked for in a task. Furthermore, comparison is also a skill that students use in their everyday life by creating categories and then making decisions [62] (p. 115). In this cognitive process, similarities and differences are identified based on criteria [63] (p. 6). Therefore, criteria are an important component of perspective-taking for students to compare the arguments of actors systematically and subsequently make a well-founded evaluation. Thus, it can be assumed that the authors consider undertaking a comparison as an everyday competency that does not require explicit tasks to be set. However, comparing is a complex cognitive process and students should be guided to implement comparison by using specific tasks [30]. Therefore, to promote the ability of perspective-taking, detailed tasks should be formulated that also address how or which criteria should be chosen.

**Dimension: Analysis**

Figure 3 shows that in 61% of the articles, the *analysis* of perspectives was generally required (implicitly or explicitly) from students. However, an explicit analysis of the perspectives was only required by the authors in 25% of the articles analysed. The students had to analyse the interests of the actors and explain the arguments of the respective perspectives regarding their differences, as in the following task: “Hypothesise and explain why the perspectives of the various actors differ from each other and derivate the potential interests of these actors based on their statements” [64] (p. 41, own translation). However, it is not clear in this example on what basis the students should explain the different perspectives of the actors. In general, an analysis must contain a targeted and systematic exploration of the existing material, which can only be achieved with criteria. Notably, only one task asked specifically for analysis of the actor’s argument by using criteria: “Analyse in partner work M2 or M3. For this purpose, create an evaluation grid based on this model […]” [65] (p. 25). In other tasks in the analysed articles, neither the criteria for analysis were given, nor were the students asked to determine their own criteria for analysing the arguments of the different actors in order to achieve a systematic processing of the material. Additionally, students rarely needed to explain the rationales (N = 1), ethical principles (N = 2) or targets (N = 2) of the respective actors. Furthermore, none of the authors required a derivation regarding the assertiveness of the actors by analysing, for example, the actor’s scale, type or category.

However, explanations of the actors’ relational, ethical principles, and targets are necessary to understand the action of each actor [29,66,67]. Additionally, the actor’s scale is an important aspect of geography in terms of the three key concepts of places, spaces and scale [68] (p. 48) because decisions that are made at one scale may have an impact on another actor’s scale, even if they were not involved in the decision-making process [69]. Therefore, respective analysis is important because it is the actors who are creating geography [70]. It is therefore even more important to analyse and understand the arguments of the actors in order to be able to participate in social discourse [71,72].

The results show that the authors largely do not consider the analysis of perspectives as an important part of perspective-taking. However, analysis is essential for the process of understanding because without such an analysis, students may not be able to make a well-founded evaluation or reflection because the similarities or differences between perspectives will not be apparent to them.

**Dimension: Deconstruction**

A point of view reflection was implemented (implicitly or explicitly) in 39% of the analysed articles, and self-reflection in 29% (see Figure 3). In the majority of the articles analysed, the authors did not intend to include a reflection of the perspectives. In only 18% of the articles was a point of view reflection explicitly required, such as in the following task: “How do you explain the attitude of one interviewee regarding the Poles?” [58] (p. 57 own translation). In this example, the students are asked to explain why the actor has this attitude and position towards the Poles. However, students do not have to explicitly question their perspective regarding affiliations to a certain social ecosystem, i.e., their interests, targets, ethical principles, assertiveness or relationships to other actors.

An explicit self-reflection was only required of the students in one task: “Read again your notes from Task 1. How did and/or didn’t your first impressions and assumptions change?” [61] (p. 38, own translation). In this example, the students were supposed to include their own perspective of the topic. However, students did not have to explicitly question their own perspective regarding their affiliation to a certain social ecosystem. Additionally, the task does not require understanding of a student’s own perspective and how it is temporally and spatially determined.

Therefore, the assumption can be made that some authors are of the opinion that the point of view reflection and self-reflection must not be explicitly required and instructed in a task, assuming that the didactic explanations or the implicit tasks are adequate. According to Rhode-Jüchtern [8] (p. 273), the deconstruction of perspectives is a necessary qualification to enable participation in a democratic society. Although it is one of the central tasks of teaching and education to teach the students to decentre themselves, it is mostly disregarded in didactics [3] (p. 18). Therefore, the point of view reflection is an important part of perspective-taking because students learn to consider other points of view, and also learn to question them.

However, point of view reflection and self-reflection are challenging skills that can overwhelm students if precise tasks are missing. Therefore, such fundamental tasks are indispensable for implementing deconstruction in the context of perspective-taking.

**Dimension: Evaluation**

Figure 3 shows that in 71% of the articles analysed, evaluation of the issue or the conflict was required (implicitly or explicitly). Explicitly, students were only asked to undertake an evaluation in 29% of the analysed articles, as in this example: “[…] in part two, make a reasoned comment regarding project ASA” [73] (p. 36, own translation). However, in only two of the 28 articles analysed did the authors differentiate between a factual judgement and a value judgement [74,75].

At this point, the importance of the distinction between a factual and a value judgement should be discussed. In a factual judgement, students choose criteria and then evaluate the arguments of the respective perspectives regarding their cogency and sufficiency. In a value judgement, students are required to evaluate the arguments of the respective perspectives based on their own ethical principles. Therefore, it is important to raise students’ awareness of the type of judgement so that there is no confusion between a factual judgement and a value judgement. If the distinction between a factual judgement and a value judgement is not made, it can lead to randomness in the argumentation [76] (p. 306). Thus, it would be important to use the action verbs *judge*, *assess* and *comment on* in a differentiated way, so that the type of evaluation is obvious for students.

Therefore, in terms of conceptual change, it can also be deduced for education sciences that students should learn whether intuitive reasoning seems appropriate or not [77,78]. In addition, scientific misconceptions could also be taken into account in order to promote the pupils’ competency to evaluate [77] (p. 180).

Moreover, in geography, there is rarely only one correct result and often no entirely correct argument. Therefore, the issue and the conflict must be discussed in an open and unbiased way. However, an argument can only be considered of high quality if it contains different perceptions of different perspectives, spatial references and a complex rationale [72] (p. 276). It is therefore important to guide the factual judgement and the value judgement with tasks so that perspectives, criteria, and arguments of the actors and their assertiveness are considered in the student’s evaluation. Thus, the students can learn to develop a high-quality argument.

If, in addition, the students disclose their own ethical principles, they can also recognise their own established beliefs. In this way, ethical principles can also be discussed and reflected upon [79] (pp. 45–48), promoting tolerance of ambiguity and democratic education [28] (p. 3).

**Dimension: Metareflection**

In 25% of the articles, an implicit metareflection was targeted by the authors (Figure 3). In most of the articles analysed, the authors did not include a metareflection of the perspectives.

Thus, it can be assumed that these authors do not consider the metareflection as an essential part of perspective-taking. It could also be possible that the authors do not consider an explicit task to be necessary or they do not want to overwhelm students. However, metareflection is an important part of perspective-taking because it shows students that each perspective is only one out of many possible perspectives. Accordingly, for example, the evaluation of an issue or conflict can change depending on the choice of the actor’s arguments, as well as the choice of criteria or their own ethical principles. This enables the students to understand that the selected perspectives and their own perspective give the conflict or the issue a specific meaning or direction. It is also the understanding that there are irreconcilable differences which can be revealed by the competency of perspective-taking in order to recognise the relativity of one’s own point of view [80] (p. 226).

With this in mind, students can understand that it is not possible to achieve an absolute evaluation that includes all existing or possible perspectives.

## 5. Conclusions

Perspective-taking is a complex process of understanding; therefore, developing it as a competency can support students in developing their social cognition as well as their critical thinking. Therefore, more precise tasks for perspective-taking could help to further promote competence-oriented geography teaching.

In the educational standards in geography, perspective-taking is considered as an important component of evaluation: students should consider a conflict or an issue from different points of view and stereotypical thinking should be prevented [4]. In this regard, students must be able to tolerate ambiguous situations in terms of tolerance of ambiguity [3] (p. 14) and deconstruct perspectives [8] (p. 273). Although perspective-taking is an important competency that supports students in avoiding a monocausal consideration and instead making a differentiated evaluation, empirical research in the field of geography education has so far hardly dealt with perspective-taking. Therefore, we analysed which competencies for perspective-taking were considered important for geography lessons by the authors of didactic geographic journals. Lesson concepts and tasks from four practice journals of geography education were analysed. The analysis was based on a developed normative competency model for perspective-taking (see Figure 1). All 28 articles analysed were focused on perspective-taking, and had been published over the last 29 years in German-speaking countries. Overall, the analysis showed that there were few geography–didactic proposals for implementing perspective-taking in geography lessons, and subsequently, these articles are only partially suitable for promoting students’ competency in perspective-taking.

In nine of the lesson concepts analysed (32%), no task could be identified that explicitly guided students on how to implement perspective-taking. The dimensions of metareflection and acknowledgement of the competency model for perspective-taking were not explicitly included in any of the authors’ developed lesson concepts. It is also noticeable that the students were rarely asked to compare perspectives systematically when undertaking a comparison, for example, by working with criteria. In addition, the authors provided little to no assistance on how the analysis of the perspectives should be implemented by the students in the tasks. How the actors’ arguments should be analysed in terms of their interests, targets, ethical principles and respective rationales were also not explained. Furthermore, the students were not asked to determine the assertiveness of the actors in any of the analysed articles. However, without a well-founded analysis, perspectives cannot be probably deconstructed in the point of view reflection and self-reflection.

In contrast, students were asked to evaluate an issue or conflict in most of the articles. However, the authors hardly made any differentiation between a factual judgement and a value judgement. In a factual judgement, students choose criteria and then evaluate the arguments of perspectives regarding their cogency and sufficiency, whereas the students evaluate the arguments of the respective perspectives based on their own ethical principles in a value judgement. This differentiation should therefore be explicitly stated as part of set tasks [50] (p. 6), because specific tasks can help students to better consider certain aspects [81] (p. 161). Detailed tasks are indispensable for perspective-taking so that students can enhance their competency. Although acknowledgement, comparison, analysis and evaluation (see Figure 3) were at least implicitly required by the authors of the analysed articles, competency reflection was rarely promoted in the analysed articles. Our competence model, derived from different concepts, theories and existing models, currently identifies which dimensions should be included in the competence for perspective-taking. The analysis of the articles should thereby show which dimensions of perspective-taking have been included in the tasks. Our model is therefore suitable for the analysis and the development of teaching materials that implement perspective-taking.

Based on this model, we can define levels for each dimension in the next step to develop and to test a competency level model in order to finally design a development model if possible. In addition, the competency level model can also be a valuable tool for diagnostics to classify students’ competency in terms of perspective-taking.

## Data Availability

The data presented in this study are available on request from the corresponding author. The data are not publicly available due to data protection and privacy.
